# Global Prevalence of Periodontal Disease and Lack of Its Surveillance

**DOI:** 10.1155/2020/2146160

**Published:** 2020-05-28

**Authors:** Muhammad Nazir, Asim Al-Ansari, Khalifa Al-Khalifa, Muhanad Alhareky, Balgis Gaffar, Khalid Almas

**Affiliations:** Department of Preventive Dental Sciences, College of Dentistry, Imam Abdulrahman Bin Faisal University, Dammam, Saudi Arabia

## Abstract

**Background:**

Periodontal disease is a public health problem and is strongly associated with systemic diseases; however, its worldwide distribution is not fully understood.

**Objective:**

To evaluate global data of periodontal disease: (1) among adolescents, adults, and older population and (2) in low-, middle-, and high-income countries.

**Methods:**

This ecological study included data of periodontal disease from the World Health Organization's data bank which are based on the Community Periodontal Index of Treatment Needs (CPITN code: 0 = no disease; 1 = bleeding on probing; 2 = calculus; 3 = periodontal pocket (PD) 4-5 mm; 4 = PD (6+ mm). Age- and income-related periodontal disease inequalities were evaluated across the globe.

**Results:**

Compared with 9.3% of adults and 9.7% of older persons, 21.2% of adolescents had no periodontal disease (*P* = 0.005). Nearly 18.8% of adolescents compared with 8.9% of adults and 5% of older persons had bleeding on probing (*P* ≤ 0.001). Similarly, 50.3% of adolescents, 44.6% of adults, and 31.9% older persons demonstrated the occurrence of calculus (*P* = 0.01). On the other hand, older persons had the highest prevalence of PD 4-5 mm and PD 6+ mm than adults and adolescents (*P* ≤ 0.001). The distribution of periodontitis (CPITN code 3 + 4) in adults differed significantly in low- (28.7%), lower-middle- (10%), upper-middle- (42.5%), and high-income countries (43.7%) (*P* = 0.04). However, no significant differences in periodontitis (CPITN code 3 + 4) were observed in adolescents and older persons in low- to high-income countries.

**Conclusions:**

Within the limitations of data, this study found that the distribution of periodontal disease increases with age. Periodontitis was the most common in older persons and in population from high-income countries.

## 1. Introduction

Periodontal disease which comprises gingivitis and periodontitis is a common oral infection that affects the tissues that surround and support teeth [1]. The condition often presents as gingivitis which is characterized by bleeding, swollen gums, and pain, and if left untreated, it progresses to periodontitis which involves the loss of periodontal attachment and supporting bone [2]. According to the Global Burden of Disease Study (2016), severe periodontal disease was the 11^th^ most prevalent condition in the world [3]. The prevalence of periodontal disease was reported to range from 20% to 50% around the world [4]. It is one of the major causes of tooth loss which can compromise mastication, esthetics, self-confidence, and quality of life [5, 6]. Globally, periodontal diseases accounted for 3.5 million years lived with disability (YLD) in 2016 [3]. During the period from 1990 to 2010, there was a 57.3% increase in the global burden of periodontal disease [7]. In 2010, worldwide loss of productivity due to severe periodontitis was estimated to be US$54 billion per year [8]. The global prevalence of periodontal disease is expected to increase in coming years due to growth in the aging population and increased retention of natural teeth due to a significant reduction in tooth loss in the older population [9].

Masticatory difficulties resulting from periodontal disease can interfere with the intake of food, thus negatively affecting nutrition and the general health of patients [5]. In addition, periodontal disease is associated with other common systemic conditions such as diabetes, cardiovascular disease, adverse pregnancy outcomes, rheumatoid arthritis, and chronic obstructive pulmonary disease [10–14]. The metastatic spread of microorganisms and their products from dental plaque and inflammatory mediators from periodontal tissues to other organs of the body is believed to account for this periodontal and systemic disease connection [14–16].

Different segments of the population are disproportionally affected with periodontal disease [17]. Evidence has suggested an inverse relationship between income and periodontal disease [18]. It was reported that low-income individuals had 1.8 times increased odds of severe periodontal disease than high-income individuals [17]. Periodontal disease inequalities exist among different age groups, and the severity of the disease increases with advancing age. In an epidemiological study, it was found that the highest prevalence of chronic periodontist was found in the elderly population (82%), followed by adults (73%) and adolescents (59%) [19]. It is known that periodontal disease can be prevented; however, patients with periodontal disease usually seek oral care when the disease reaches an advanced stage because its early stages are usually asymptomatic [20]. Therefore, early diagnosis and treatment are crucial for the maintenance of periodontal health.

The analysis of global data about the prevalence of periodontal disease is useful for policy development and the allocation of financial and human resources for preventive measures and the provision of treatment. However, the prevalence of periodontal disease in different age groups and in low-income, middle-income, and high-income countries is not fully understood. Therefore, this study aimed to compare global data of periodontal disease among population of adolescents, adults, and older persons. The study also evaluated the prevalence of periodontal disease in low through high-income countries.

## 2. Methods

Globally, there are discrepancies in the prevalence of periodontal disease in epidemiological studies due to the differences in sample size, sampling technique, disease measurement method/diagnostic technique, definitions of periodontal disease, socioeconomic conditions of study population, and timing of study [21, 22]. However, the use of a universally accepted diagnostic method remains one of the challenges in epidemiological investigations of periodontal disease [22]. Although periodontal disease is frequently diagnosed by probing or loss of attachment, patient discomfort due to pain on probing, long time for examination, and the possibility of the spread of infection are concerns in population studies [23]. The World Health Organization (WHO) proposed the use of community periodontal index (CPI), a needs assessment tool for the planning of resources in 1977. The extensive use of CPI around the world helped obtain comparable data to evaluate global distribution and trends of periodontal disease [24].

The WHO collaborates with many organizations and individuals worldwide to collect information about oral conditions and oral health services and maintains under the WHO oral health country/area profile programme (CAPP). For this study, data were retrieved from the WHO oral health data bank about periodontal health profile of countries that were maintained and updated by the Niigata University Graduate School of Medical and Dental Sciences in Japan [25]. The WHO periodontal disease data are based on scores of CPI for adolescents (15–19 years), adults (35–44 years), and older persons (65–74 years). CPI score ranges from 0 to 4 and a score 0 = healthy periodontal conditions or no periodontal disease; score 1 = gingival bleeding; score 2 = calculus and bleeding; score 3 = shallow periodontal pockets (4–5 millimeters); and a score 4 = deep periodontal pockets (6 millimeters or more) [26].

Periodontal Country Profile data from 2000 to 2016 were used for our analysis. Data between 1981 and 1999 were excluded from the study to provide more recent estimates of periodontal disease. Periodontal disease data were compared among adolescents, adults, and older persons in selected countries [25]. The World Bank (2017) categorizes countries into low-income, lower-middle-income, upper-middle-income, and high-income. Low-income countries had gross national income (GNI) per capita ≤ $995; lower-middle-income countries had GNI per capita $996 and $3,895; upper-middle-income countries had GNI per capita between $3,896 and $12,055; and high-income countries had GNI per capita ≥ $12,056 [27].

Statistical Package for Social Science (SPSS Statistics for Windows, Version 22.0, Armonk, NY: IBM Corp) was used for statistical analysis. CPITN (codes 3 and 4) was combined as indicators of periodontitis to present disease distribution among adolescents, adults, and older population. The Kruskal–Wallis test was performed to compare periodontal disease data among adolescents, adults, and older population in selected countries. Similar comparisons were made among low-income, lower-middle-income, upper-middle-income, and high-income countries. A *P* value of ≤0.05 was considered statistically significant.

## 3. Results

**Figure 1:**
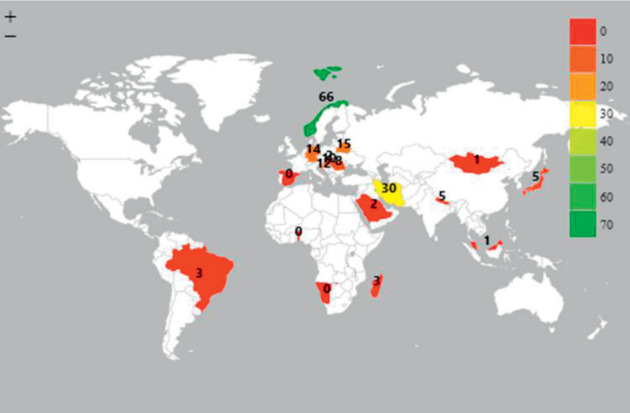
Prevalence of periodontitis (CPITN code 3 + 4) among adolescents (15–19 years).

The study analyzed data of adolescents (15–19 years), adults (35–44 years), and older persons (65–74 years) from 27 low to high income countries. Belarus had the highest prevalence of periodontal disease among adolescents because there was no adolescent without periodontal disease (0 percent of adolescents with no disease CPITN Code = 0). This was followed by Norway (1% with no periodontal disease) and Germany with 2% of adolescents with no periodontal disease. Periodontitis (CPITN code 3 + 4) in adolescents was most common in Norway (66%), followed by Iran (30%) and Belarus (15%). Germany and Taiwan had 14% of their adolescents with periodontitis (CPITN code 3 + 4) (Figure 1).

**Figure 2:**
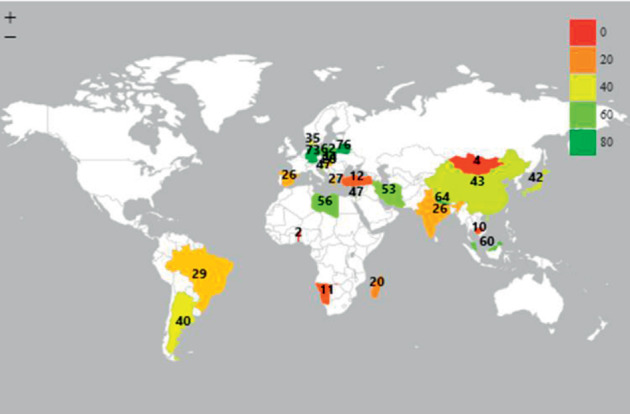
Prevalence of periodontitis (CPITN code 3 + 4) among adults (35–44 years).

Two most populated countries in the world, China and India, had no adult without periodontal disease (0 percent of adults with no disease CPITN code = 0). In addition, Belarus had no adults without periodontal disease, while Germany and Taiwan had 1% of adults with no disease. Adults in Belarus (76%), Germany (73%), and Nepal (64%) demonstrated the highest prevalence of periodontitis (CPITN code 3 + 4). More than half of adult population in Poland (62%), Malaysia (60%), Libya (56%), Iran (53%), and Taiwan (53%) had periodontitis (CPITN code 3 + 4) (Figure 2).

**Figure 3:**
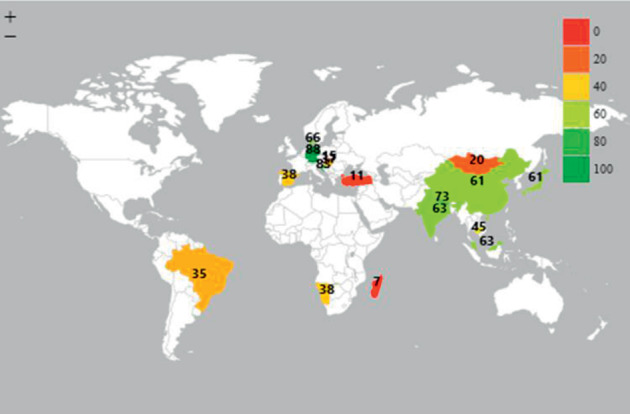
Prevalence of periodontitis (CPITN code 3 + 4) among older persons (65–74 years).

Hundred percent of older persons in China, India, and Croatia have periodontal disease (0 percent of older persons with no disease CPITN Code = 0). The highest prevalence of periodontitis (CPITN code 3 + 4) in older persons was found in Germany (88%), Croatia (83%), Nepal (73%), and Taiwan (73%) (Figure 3).

**Figure 4:**
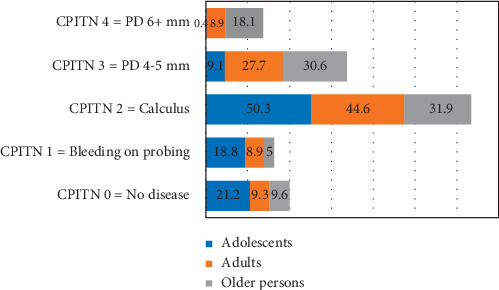
Global distribution of periodontal disease in adolescents, adults, and older persons.

Figure 4 shows the overall prevalence of periodontal disease in adolescents, adults, and older persons. The presence of calculus is the most common in adolescents, adults, and older persons, and this is followed by the occurrence of PD 4-5 mm. There were statistically significant differences in the prevalence of no disease, bleeding on probing, calculus, PD 4-5 mm, and PD 6 + mm among adolescents, adults, and older population. The adolescents had the highest prevalence of no periodontal disease (21.2%) compared with adults (9.3%) and older population (9.7%) (*P*=0.005). On the other hand, 18.8% of adolescents compared with 8.9% of adults and 5% of older persons had bleeding on probing (*P* ≤ 0.005). Similarly, half the adolescents (50.3%), adults (44.6%), and older persons (31.9%) demonstrated calculus (*P*=0.01). PD 4-5 mm and PD 6 + mm were highly distributed in older persons than adults and adolescents and differences were significant (*P* ≤ 0.001).

Significant differences were observed regarding bleeding on probing (*P*=0.018) when data were compared among low-income (3.12%), lower-middle-income (9.77%), upper-middle-income (10.05%), and high-income countries (13.96%). Calculus was the most commonly distributed among lower-middle-income countries (58.66%), and differences were significant (*P*=0.028).

**Figure 5:**
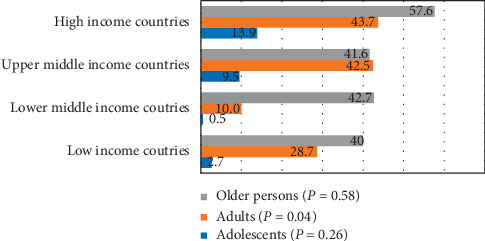
Distribution of periodontitis (CPITN code 3 + 4) in low-income, lower-middle-income, upper-middle-income, and high-income countries.

Lower-middle-income countries had the lowest distribution (0.5%) of periodontitis (CPITN code 3 + 4) and high-income countries had the highest distribution of disease (13.9%), and differences were not significant (*P*=0.26). However, there were significant differences in the distribution of periodontitis (CPITN code 3 + 4) in adults in low- (28.7%), lower-middle- (10%), upper-middle- (42.5%), and high-income countries (43.7%) (*P*=0.04). The distribution of periodontitis (CPITN code 3 + 4) in older persons did not differ significantly in low-income to high-income countries (*P*=0.58) (Figure 5).

## 4. Discussion

The present study demonstrated that the global prevalence of periodontal disease increases with age from adolescents to adults and older population. It was also found that CPI 3 (PD 4-5 mm) and CPI 4 (PD 6 + mm) were highly concentrated among older people. A previous WHO questionnaire-based study from 46 countries found that periodontal disease, reflected by CPI 3 and CPI 4, was the most frequent in older population [28]. Similarly, poor periodontal health in older people has been previously illustrated in Indonesia, and it was reported that there was a significant correlation (coefficient correlation = 0.251, *P* ≤ 0.001) between the age of older persons and periodontal disease [19]. Data from National Health and Nutrition Examination Surveys in the U.S. showed that 40.7% of 65 years and older population experienced attachment loss of ≥6 mm, and 22.7% demonstrated periodontal pockets ≥5 mm [29]. A review of 75 studies reported that the prevalence of severe periodontitis increases with age and peaks at the age of 40 years and then remains stable in older age, hence exhibiting a high burden of disease in the elderly population [30]. An epidemiological study in Sweden found that the proportion of subjects with pocket depths of more than 4 mm increased with age [31]. The severity of periodontal disease increases with advancing age, and similar patterns of occurrence of disease were reported in several studies [32–36].

High prevalence of periodontal disease in older population can be attributed to poor oral hygiene, lack of government financing for oral health services, and lack of oral health promotion programs and policies aimed at the older population in various countries around the world [28]. In addition, the high concentration of periodontal destruction in older people could be because of the cumulated effect of untreated periodontal disease over a period of time rather than the effect of age on periodontal disease [37]. Aging is known to impair the immune and inflammatory responses which contribute to periodontal tissue destruction in older subjects [38].

In the present study, disparities in the severity of periodontal disease demonstrated by CPI 3 and CPI 4 existed in low-, middle-, and high-income countries. It was found that high-income countries had the highest prevalence of CPI 3 (PD 4-5 mm) and CPI 4 (PD 6 + mm). Globally, the number of older persons increased from 382 million in 1980 to 962 million in 2017, and it is projected to increase to 1.4 billion by 2030 [39]. Similarly, the older population has increased dramatically during the last four decades particularly in high-income countries (European and North American countries) compared with low-income countries (African and Asian) [39]. Twenty-five percent of European population was over the age of 60 years or over in comparison with 5% of African population in 2017 [39]. Older population makes a considerable segment of the society in high income countries that may account for the increased occurrence of periodontal pockets in these countries [28].

Empirical research has shown an inverse relationship between the severity of periodontal disease and individual income [18]. Borrell et al. indicated that low-income subjects had significantly higher odds of (odds ratio = 1.8) having severe periodontal disease than high-income subjects [17]. Similarly, the report of the third National Health and Nutrition Examination Survey (NHANES III) in the U.S. showed that individuals living in the low socioeconomic neighborhood were 1.81 times more likely to have periodontitis than those living in the high socioeconomic neighborhood [40]. The Korean National Health and Nutrition Examination Survey IV (2007-209) also found similar trends of increased periodontal disease among low-income individuals [41]. The literature has consistently shown inequalities about periodontitis among individuals of varying income backgrounds [42, 43].

Low income is one of the barriers to access to oral health care. The utilization of dental services is related to the availability of dental insurance. It is documented that individuals with dental insurance perform more routine dental visits than those without dental insurance [44]. Similarly, low-income individuals may have low perception about the importance of oral health or may not be fully aware of the need for dental care and may also have low expectation of good health [45]. Therefore, individuals from high-income segments of the society compared with low income people are more likely to have dental insurance and receive both preventive and curative dental care. These factors contribute to the retention of natural teeth among high-income individuals [9].

High distribution of periodontal disease particularly CPI 3 and CPI 4 in high-income countries in the present study can be explained by the exponential growth in the aging population and increased retention of natural teeth among individuals in these countries. Policy-makers, public health professionals, and stakeholders should consider the effect of increased life expectancy and retention of natural teeth when developing policies and programs to improve periodontal health, particularly in high-income countries. They should integrate oral health programs into national health programs as emphasized by the World Health Organization [28].

Periodontal disease is a global public health problem. There is a dramatic increase in the burden of periodontal disease during the last decades, and a large body of evidence shows its strong significant association with systemic diseases; however, limited periodontal data are available in the WHO oral health data bank. Oral health programs aimed at preventing periodontal disease require robust epidemiological data, and the allocation of health resources to provide treatment for periodontal disease cannot be achieved in the absence of updated and reliable data. Of 193 countries of the United Nations, periodontal disease data of only 20 countries for adolescents, 27 countries for adults, and 18 countries for older persons were maintained by the WHO. Even data of a few countries were collected during the last five years. Moreover, there was no continuous and systematic collection of epidemiological data from different countries. This lack of periodontal disease surveillance at the global stage calls for integrated actions from public health professionals, researchers, periodontologists, and local, national, and global health organizations.

Empirical research does provide important information; however, an ecological comparison among countries shows meaningful trends and patterns of periodontal disease. The study included data of periodontal disease based on the CPITN which provided reliable and valid comparisons and valuable information for stakeholders. The CPITN instrument is commonly used in epidemiological studies due to its wider acceptance among researchers. However, the CPITN uses probing depth as the main clinical parameter to measure periodontitis which is known to overestimate the prevalence of periodontitis. Hence, the instrument may not show actual severity and extent of periodontal disease in a screened population [46]. The inclusion of data from 2000 till 2016 provided as recent information as possible. However, the exclusion of periodontal disease information between 1981 and 1999 limits the generalizability of study findings. In addition, data were collected from various countries at different points in time. Therefore, the study findings call for establishing a surveillance system for periodontal disease by continually collecting national representative data from most countries around the world.

## 5. Conclusion

Within the limitations of data, the study showed that periodontal inequalities existed in different populations around the globe. Periodontal disease was the most common among older population. Adolescents in selected countries more frequently demonstrated bleeding on probing than adults and older persons. Periodontal pockets (PD 4-5 mm and PD 6 + mm) were disproportionally and highly distributed among older persons. Significant differences regarding bleeding on probing existed among low-income, middle-income, and high-income countries. Low- and middle-income countries had higher occurrence of calculus than high-income countries. The prevalence of periodontal pockets was the most frequent in high-income countries.

## 6. Recommendations

- The WHO through collaborations should establish surveillance of periodontal disease to systemically collect epidemiological information in a similar way as it is obtained for many systemic diseases from most countries
- Funding should be provided to conduct national surveys in low-income countries
- High prevalence of periodontal disease in rapidly progressing older population warrants the integration of periodontal disease prevention programs and policies into general health preventive initiatives
- The development of integrated oral and systemic health policies should commence at local, national, and international levels

## Data Availability

The SPSS data file of this study is available from the corresponding author upon request.

## Supplementary Materials

Supplementary Materials Table 1: prevalence of periodontal disease among adolescents (15–19 years); Table 2: prevalence of periodontal disease among adults (35–44 years); Table 3: prevalence of periodontal disease among older persons (65–74 years).
