# Supplementary material for: Global Prevalence of Periodontal Disease and Lack of Its Surveillance
Source: ScientificWorldJournal. 2020 May 28;2020:2146160. doi: 10.1155/2020/2146160 (PMC7275199; doi:10.1155/2020/2146160)
Supplement: Supplementary Materials — Table 1: prevalence of periodontal disease among adolescents (15–19 years); Table 2: prevalence of periodontal disease among adults (35–44 years); Table 3: prevalence of periodontal disease among older persons (65–74 years). [file 2146160.f1.pdf]

Supplementary files

Table 1. Prevalence of periodontal disease among adolescents (15-19 years)

Countries	Year of study	0 (No disease) (%)	1(Bleeding on probing) (%)	2 (Calculus) (%)	3 (PD 4-5mm) (%)	4 (PD 6+mm) (%)
**Croatia**	2000	16	20	52	12	0
**Germany**	2005	2	51	33	13	1
**Hungary**	2003	36	19	35	10	0
**Japan**	2011	31	24	41	5	0
**Norway**	2003	1	19	13	58	8
**Saudi Arabia**	2010	14	24	61	2	0
**Slovakia**	2000	23	24	51	2	0
**Spain**	2005	35	37	29	0	0
**Taiwan**	2008	3	15	68	14	0
**Comoros**	2001	8	1	90	0	0
**Mongolia**	2012	30	24	46	1	0
**Benin**	2003	46	1	53	0	0
**Madagascar**	2002	19	6	66	3	0
**Nepal**	2004	26	8	61	5	0
**Belarus**	2000/2001	0	7	79	15	0
**Brazil**	2005	48	40	10	3	0
**Iran**	2004	11	12	46	30	0
**Malaysia**	2007	11	13	75	1	0
**Namibia**	2013	39	17	44	0	0
**Romania**	2009	26	14	53	8	0
**Total**		21.25	18.8	50.3	9.1	0.45

Table 2. Prevalence of periodontal disease among adults (35-44 years)

**Countries**	**Year of study**	**0 (No disease) (%)**	**1(Bleeding on probing) (%)**	**2 (Calculus) (%)**	**3 (PD 4-5mm) (%)**	**4 (PD 6+mm) (%)**
**Argentina**	1999/2000	3	14	43	26	14
**Croatia**	2000	6	6	41	32	15
**Denmark**	2000/01	8	16	41	29	6
**Germany**	2005	1	12	14	52	21
**Greece**	2005	10	16	47	24	3
**Hungary**	2003	11	5	57	22	6
**Israel**	2008/2009	5	10	38	33	14
**Japan**	2016	29	11	18	37	5
**Poland**	2011	5	15	18	45	17
**Slovakia**	2000	8	5	44	29	15
**Spain**	2005	15	13	47	22	4
**Taiwan**	2008	1	2	44	37	16
**Cambodia**	2011	4	4	80	8	2
**Comoros**	2000/01	2	0	84	0	0
**Mongolia**	2012	30	7	59	3	1
**India**	2011	0	37	37	26	0
**Benin**	2003	37	1	61	1	1
**Madagascar**	2002	8	5	67	17	3
**Nepal**	2004	7	3	27	48	16
**Belarus**	2000/2001	0	0	24	63	13
**Brazil**	2010	26	3	42	23	6
**China**	2012	0	0	57	30	13
**Iran**	2002	1	6	40	43	10
**Libya**	2012	2	4	38	47	9
**Malaysia**	2010	2	2	36	35	25
**Namibia**	2013	20	15	54	8	3
**Turkey**	2010	11	30	47	9	3
**Total**		9.33	8.96	44.63	27.7	8.92

Table 3. Prevalence of periodontal disease among older persons (65-74 years)

Countries	Year of study	0 (No disease) (%)	1(Bleeding on probing) (%)	2 (Calculus) (%)	3 (PD 4-5mm) (%)	4 (PD 6+mm) (%)
**Croatia**	2000	0	0	17	48	35
**Denmark**	2000/01	2	9	23	46	20
**Germany**	2005	1	4	7	48	40
**Hungary**	2003	7	10	46	26	11
**Japan**	2016	23	6	10	44	17
**Slovakia**	2000	63	4	18	11	4
**Spain**	2005	10	13	39	27	11
**Taiwan**	2008	1	1	26	46	27
**Cambodia**	2011	2	1	52	24	21
**India**	2011	0	0	37	37	26
**Mongolia**	2012	24	14	43	16	4
**Madagascar**	2002	1	0	17	5	2
**Nepal**	2004	1	1	18	48	25
**Brazil**	2010	19	2	44	26	9
**China**	2012	0	0	39	33	28
**Malaysia**	2010	3	2	32	33	30
**Namibia**	2013	3	4	51	24	14
**Turkey**	2005	14	20	55	9	2
**Total**		9.66	5.05	31.89	30.61	18.11
